# The Optimization of Avocado-Seed-Starch-Based Degradable Plastic Synthesis with a Polylactic Acid (PLA) Blend Using Response Surface Methodology (RSM)

**DOI:** 10.3390/polym16162384

**Published:** 2024-08-22

**Authors:** Rozanna Dewi, Novi Sylvia, Zulnazri Zulnazri, Herman Fithra, Medyan Riza, Januar Parlaungan Siregar, Tezara Cionita, Deni Fajar Fitriyana, Samsudin Anis

**Affiliations:** 1 Chemical Engineering Department, Malikussaleh University, Lhokseumawe 24353, Aceh, Indonesia; novi.sylvia@unimal.ac.id (N.S.); zulnazri@unimal.ac.id (Z.Z.); 2 Center of Excellence Technology Natural Polymer and Recycle Plastics, Malikussaleh University, Lhokseumawe 24353, Aceh, Indonesia; 3 Civil Engineering Department, Malikussaleh University, Lhokseumawe 24353, Aceh, Indonesia; hfithra@unimal.ac.id; 4 Chemical Engineering Department, Syiah Kuala University, Banda Aceh 23111, Aceh, Indonesia; medyan_riza@usk.ac.id; 5 Faculty of Mechanical and Automotive Engineering Technology, Universiti Malaysia Pahang Al-Sultan Abdullah, Pekan 26600, Pahang, Malaysia; januar@ump.edu.my; 6 Department of Mechanical Engineering, Faculty of Engineering and Quantity Surveying, INTI International University, Seremban 71800, Negeri Sembilan, Malaysia; tezara.cionita@newinti.edu.my; 7 Department of Mechanical Engineering, Universitas Negeri Semarang, Kampus Sekaran, Gunungpati, Semarang 50229, Central Java, Indonesia; deniifa89@mail.unnes.ac.id (D.F.F.); samsudin_anis@mail.unnes.ac.id (S.A.)

**Keywords:** avocado seed starch, PLA, RSM, degradable plastic

## Abstract

This research improves the strength of plastic using avocado seed starch and PLA. The effect of blending avocado seed starch and PLA was optimized using the RSM approach by using two variables: water absorption and biodegradability. Mixing them using RSM gave the best result: 1.8 g of starch and 3 g of PLA. Degradable plastic has a tensile strength of 10.1 MPa, elongation at a break of 85.8%, and a Young’s modulus of 190 MPa. Infrared spectroscopy showed that the plastic had a -OH bond at 3273.20 cm^−1^, 3502.73 cm^−1^, and 3647.39 cm^−1^, a CH_2_ bond at 2953.52 cm^−1^, 2945.30 cm^−1^, and 2902.87 cm^−1^, a C=C bond at 1631.78 cm^−1^, and a C-O bond at 1741.72 cm^−1^. The plastic decomposed in the soil. It was organic and hydrophilic. Thermal tests demonstrated that the plastic can withstand heat well, losing weight at 356.86 °C to 413.64 °C, forming crystals and plastic melts at 159.10 °C—the same as PLA. In the melt flow test, the sample melted before measurement, and was therefore not measurable—process conditions affected it. A water absorption of 5.763% and biodegradation rate of 37.988% were found when the samples were decomposed for 12 days. The starch and PLA fused in the morphology analysis to form a smooth surface. The RSM value was close to 1. The RSM gave the best process parameters.

## 1. Introduction

In recent years, the production of plastics from biodegradable natural resources has attracted significant attention. The demand for plastic from petroleum is growing. It is non-biodegradable and made with dangerous chemicals. Waste is a serious problem, especially in crowded countries. Degradable waste from many sources harms the environment. It takes a lot of work to manage this waste. In the last decade, new biodegradable plastics have been developed [1]. Plastic harms the environment and its health because it does not break down [2]. Indonesia is the second-largest contributor to plastic waste, especially in marine areas [3]. This causes problems in the sea, land, and air [4].

Natural polymers are a great choice that requires further innovation for structural and mechanical properties comparable to conventional plastics. Therefore, plasticizing agents and fillers help minimize these conditions [5]. There have been studies on some sustainable biopolymers that are already commercially available, such as polylactic acid (PLA), polybutylene succinate (PBS), or polyhydroxyalkanoate (PHA) [6]. PLA exhibits favorable mechanical attributes, including a minimal haze or turbidity, defined as the cloudiness of a fluid and quantified as a percentage of light deflection, and an elevated tensile strength, representing the maximum stress a polymer can withstand before rupture under tensile load, expressed in MPa [7]. One solution to the problem of plastic waste is to use plastics that can be decomposed by nature in a short time and are naturally based, such as starch from fruit waste. The usage of plasticizers, blend making, and reinforcing bioplastics with lignocellulose-based materials has been shown to be a potentially promising and eco-safe option to overcome the limitation of bioplastics, mainly because of the availability, biodegradability, and biocompatibility of such materials [8].

To study and optimize the interaction effects of the process parameters, response surface methodology (RSM) was utilized [9]. The failure of various studies is frequently attributed to the presence of non-optimum operating conditions. Response surface methodology (RSM) is one approach to enhancing process conditions. This method allows for the examination of two or more factors at multiple levels while providing reliable findings across a broad range of experimental conditions. This study aimed to optimize process parameters, starch and PLA content, and their interaction effects on two fundamental properties of degradable plastics: water absorption and biodegradability. This was achieved through the implementation of RSM. The optimal results of the RSM were analyzed regarding the mechanical, chemical, thermal, and morphological properties of the degradable plastics. Mathematical and statistical tools were used to model and optimize. Validation tests were also performed to check the models. RSM-based CCD is an effective tool for predicting the relationship between input parameters and the desired response. Nwuzor et al., (2023) studied polyethylene with cassava starch blends, obtaining optimal conditions on mechanical properties and biodegradation rates [10]. Yusoff et al., (2021) developed a composite bioplastic based on PLA combined with tapioca starch (TS). From the results, the tensile strength increased significantly with the addition of TS 30 wt.% and gradually decreased with the addition of TS [11]. Ayyubi et al., (2022) studied how the properties of chitosan, cassava starch, and PVA affect biodegradable plastic using response surfaces methodology and a Box-Behnken design. They varied the chitosan, starch, and PVA amounts between 1 and 3 g, 1 and 5 g, and 1 and 5 g, respectively. The best tensile strength and elongation were found with 3 g chitosan, 1 g starch, and 5 g PVA. After 30 days in soil, it degraded by 50%. Further research could investigate the plastic’s characteristics [12].

This research studies the optimization of avocado seed starch and PLA-based degradable plastic manufacturing with RSM. The degradable plastic preparation used RSM to determine the optimal operating conditions and material composition with a central composite design (CCD) modeling software design expert 13. Yang et al., (2023) studied cellulose triacetate fiber-based reverse osmosis membranes using RSM to find optimal preparation conditions and permeability results [13]. Magesh et al., (2022) optimized and produced bioplastics from natural waste using RSM. Various parameters such as substrate concentration, temperature, pH, and time duration were optimized using RSM to improve bioplastic production [14]. In this study, the type of starch used was avocado seed starch with a mass ratio of 1.5, 2.5, and 3.5 g. Avocado seed is commonly considered agricultural waste and a potential source of pollution. This by-product, though, has excellent potential for obtaining non-conventional flour and starch. The avocado seed flours of Hass and Landrace cultivars contain high lipid and protein components to be addressed [15]. This study also used PLA as a matrix modified with maleic anhydride (MA). The variations of PLA used in this study are 3, 5, and 7 g. PLA is a degradable polymer that is recyclable, compostable, biocompatible, and renewable. PLA is the most developed and researched commercially available biopolymer [16]. The weight variation of avocado seed starch and PLA was used to obtain optimal operating conditions and composition and determine the best characteristics of degradable plastics.

## 2. Materials and Methods

### 2.1. Materials

Avocado seeds were taken from the fruit market of Lhokseumawe City, Banda Sakti District, and the starch was processed from the yield. Aquadest was taken from PT Bratachem, Surabaya, Indonesia. Polylactic acid (PLA) (C_3_H_6_O_3_) 90.08 g/mol was from Sigma-Aldrich–38534–1G, St. Louis, MO, USA and maleic anhydride (MA) 99% was from Sigma-Aldrich-8.00408.1000 and Xylen Merck Supelco 108297, Burlington, MA, USA.

### 2.2. Methods

This research was conducted using avocado seed starch and PLA mass. The mass was designed using RSM with Design Expert 13 software. The research procedure consisted of several stages: making starch from avocado seeds, thermoplastic starch, degradable plastic synthesis, and testing the resulting plastic.

#### 2.2.1. Optimization Using RSM

RSM was used to model and analyze the factors of a specific problem. It shows excellent potential in optimizing responses by analyzing how multiple variables affect them using linear or polynomial functions [17]. This study indicates that RSM is an effective tool for optimizing operating variables and effectively reducing experimentation time and costs [18]. This research was conducted using starch and PLA mass design using the RSM method with Design Expert 13 software. The mass of starch (g) and the mass of PLA (g) affect the water absorption and biodegradability of the resulting degradable plastic. Degradable plastic preparation using a central composite design (CCD) modeling design of design expert 13 software with RSM was used to determine the optimal operating conditions and material composition for the first step of the preparation process. Design-Expert software helps plan and run optimized experiments. These experiments may investigate processes, mixtures, or combinations of factors and components. The Design-Expert program facilitates the identification of statistically significant outcomes and the construction of optimal models. It has a selection of graphs that help identify salient effects and visualize results. Process condition variables were X1 = starch (g), X2 = PLA (g), and response variables were Y1 = water absorption (%), Y2 = biodegradability (%).

#### 2.2.2. Degradable Plastic Synthesis

Avocado seeds were cleaned of excess pulp, peeled to separate the skin, and washed thoroughly. They were reduced in size with a knife, water was added, and then they were blended. Then, filtering was carried out using a filter cloth. The squeeze results were precipitated for 24 h and dried in an oven for 24 h at a temperature of 50 °C. After drying, the mixture was sifted with mesh size 80. Each avocado seed starch was weighed with a particular variation and 200 mL of distilled water was added into a beaker glass. Then, it was heated and stirred to gel at a gelatination temperature of 75 °C. it was stirred until homogeneous into a copolymer, and put into an oven at 100 °C for 6 h. The dried thermoplastic starch was then crushed into smaller sizes, and 10 mL xylene was added to 0.5 g MA and PLA according to certain variations and stirred until homogeneous with a temperature of 200 °C. Furthermore, thermoplastic starch was stirred until homogeneous and then molded using a hot press for 10 min with a temperature of 105 °C.

### 2.3. Characterization and Testing

The combination of independent variable levels that can provide optimal response values was determined to be 1.8 g starch and 3 g PLA. In addition, the characteristics of the degradable plastic produced under these optimal conditions were tested.

#### 2.3.1. Water Absorption 

The degradable plastic’s water resistance was analyzed using the swelling test. The swelling degree was determined following ASTM D2765-16 [19]. The sample was weighed and then put into the solvent for 24 h. It was weighed again when swelled, then dried and weighed to obtain the final weight. Equation (1) determines the swelling degree.

(1) $$\mathrm{Swelling}\;\mathrm{Degree}\;(\%)=\frac{\mathrm{Weight}\;\mathrm{of}\;\mathrm{Expanded}\;\mathrm{Sample}\;-\;\mathrm{Weight}\;\mathrm{of}\;\mathrm{Pre}\;\mathrm{Sample}\;}{\mathrm{Weight}\;\mathrm{of}\;\mathrm{Pre}\;\mathrm{Sample}}\;\times 100\%$$

The water resistance of the degradable plastics was tested using a swelling test, characterized by the percentage swelling of the degradable plastics in water. The percent swelling analysis assesses the amount of water absorbed, which leads to the expansion of the degradable plastic.

#### 2.3.2. Biodegradability Rate 

The microbial degradation rate was analyzed by burial in soil (biodegradability). The biodegradability analysis followed the ASTM G21-13 [20] standard with the method of bioplastic in direct contact with soil. The degradable plastic sample was cut into 5 × 2 cm size. The sample was weighed as the pre-mass. The sample was put into the soil at 30 cm depth and checked every four days. The sample was removed from the soil and any remaining soil was removed. The sample was weighed again as the final mass (M_1_). The biodegradability of degradable plastic was determined using the Equation (2):

(2) $$\mathrm{Biodegradability}\;(\%)=\frac{M_{0}-M_{1}\;}{M_{1}}\;\times 100\%$$

where M_0_ is the pre-mass (g) and M_1_ is the last mass (g).

Biodegradation tests were conducted to determine the degradation rate of degradable plastics and determine the time it took for the plastic to decompose in the soil.

#### 2.3.3. Mechanical Properties 

The mechanical characterization of avocado seed starch degradable plastic using PLA was determined by tensile, elongation, and Young’s modulus analysis. The tensile strength was measured by using the ASTM D638-14 [21] (American Standard Testing and Materials) procedure. This test procedure involved using a dumbbell specimen with a 50 mm gauge length. The tensile strength and elongation of the 165 mm cut pieces were analyzed using a Mechanical Universal Testing Machine. Tensile strength and elongation could be obtained from Equations (3) and (4):

(3) $$\sigma =\frac{F_{\max}}{A}$$

where σ is tensile strength (MPa), F_max_ is maximum stress (N), and A is the cross-sectional area of the film under stress (mm^2^):

(4) $$\sigma =\frac{△l}{l_{0}}$$

where ɛ is strain (MPa); l is gauge length, the measuring length of the test specimen after elongation (mm^2^); and l_0_ is the measured length of the initial sample (mm^2^).

In this research, the material’s tensile strength was determined using a texture analysis tool. The tensile strength is the maximum ability to resist external forces before the degradable plastic is deformed or breaks. Breakage can occur by cracking due to overstress or possibly by the deformation of the structure.

#### 2.3.4. Chemical Characterization 

To find the chemical bonding in organic materials, polymers, metals, and various materials, Fourier Transform Infrared Spectroscopy (FTIR) can be widely used. FTIR used type (Shimadzu-8400S), Shimadzu, Japan. The FTIR analyzing method uses infrared rays to scan the test sample and observe chemical properties. A material is infrared-irradiated; the infrared radiation it absorbs commonly puts the molecules in a higher vibration condition. FTIR is used in this study to determine the components of the materials. The wavelength that the sample absorbs is characteristic of its molecular structure. FTIR analysis in this study was carried out at wave numbers 550–4000 cm^−1^.

#### 2.3.5. Thermal Properties 

The thermal degradation of polymer is the molecular decomposition and removal of a hydrogen atom from the polymer chain due to exposure to excessive heat. Thermal degradation serves as an upper limit for the operating temperature of the polymer—the thermal stability of degradable plastics measured by thermogravimetry analysis (TGA) (Model TGA50 SrC30025100553), New Orleans, LA, USA. The thermal stability of degradable plastics is affected by the effects of polymer heating during physical changes (glass transition and melting) by TGA. Differential scanning calorimetry (DSC) tests the thermal properties of degradable plastics. It analyzes how polymers change when heated. DSC is an effective analytical tool that enables the characterization of the physical properties of polymers. It measures heat characteristics like glass transition temperature (Tg), melting temperature (Tm), crystallization temperature (Tc), cold crystallization temperature (Tcc), melting enthalpy (∆Hm), and crystallization enthalpy (∆Hc). This study uses METTLER STARe SW 11.00, Columbus, OH, USA. Scans of approximately 10 mg of degradable plastic avocado seed starch were recorded using DSC at 10 °C/min from 30–280 °C. In the meantime, the flow rate and melting point of degradable plastics were measured using the melt flow rate (MFR) method. The MFR was utilized by the CEAST Model 7026.000, Karlsruhe, Germany, which was tested against standards established by ASTM D1238-23 [22] at a temperature of 190 °C and a load of 5 kg.

#### 2.3.6. Morphological Properties

Scanning electron microscopy (SEM) is used to study a material’s surface, structure, and cross-section. It produces images of surfaces by scanning them with a focused beam of electrons, which interact with atoms on the surface. SEM can produce high-resolution images. The sample is observed in a controlled liquid environment. This study used the JEOL SEM with an acceleration voltage of 15 kV at 40×, 1000×, 2000×, 3000×, and 6000× magnification (1 area).

## 3. Results

The results of this research, as evidenced by the data obtained on the absorption of water and the biodegradability of degradable plastics produced by analyzing mechanical properties, functional groups, thermal analysis, and morphology analysis, are as follows. Table 1 shows the data from the RSM research results using design expert central composite design (RSM-CCD) V.13 software.

This research employed RSM with design expert 13 software. The objective was to investigate the influence of PLA and cellulose mass on water absorption and biodegradability.

### 3.1. Water Absorption Result

Table 2 presents the validation data set for the experimental and predicted response values determined by RSM. Table 3 shows the ANOVA study for responses and presents the results of the water resistance tests of degradable plastics using RSM-CCD with design expert 13 software. From the research results that have been obtained, the effect of avocado seed starch and PLA on water absorption (%) in degradable plastics will be discussed.

**Figure 1:**
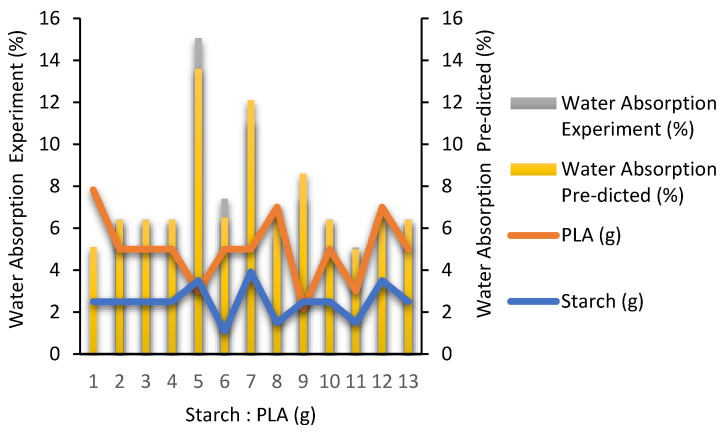
Validation data set for experimental and predicted water absorption of responses determined by RSM.

**Figure 2:**
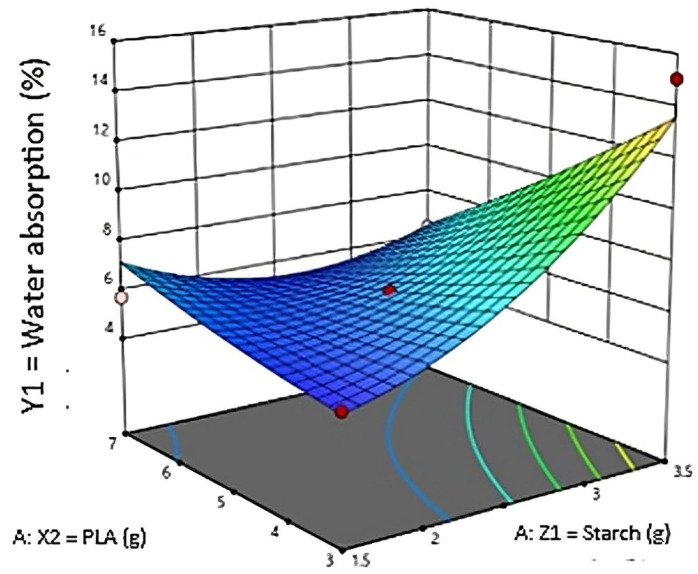
3D plot for water absorption.

Figure 1 explains validation data set for experimental and predicted water absorption of responses determined by RSM, meanwhile statistical regression of water absorption analyzed by RSM is shown in Table 4. The 3D model for water absorption is presented in Figure 2.

### 3.2. Biodegradability Rate Result

**Figure 3:**
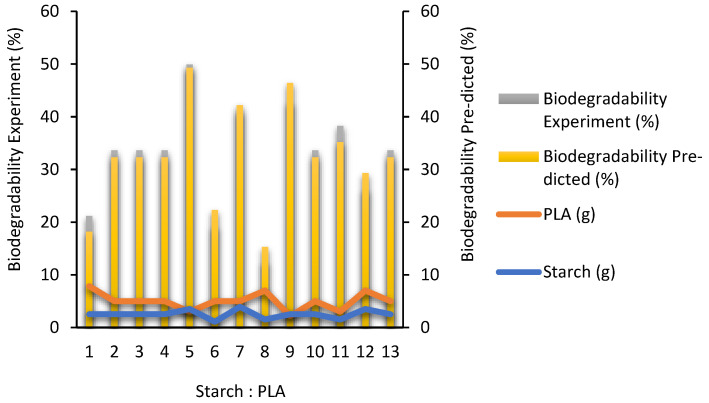
Validation data set for experimental and predicted biodegradability of responses determined by RSM.

**Figure 4:**
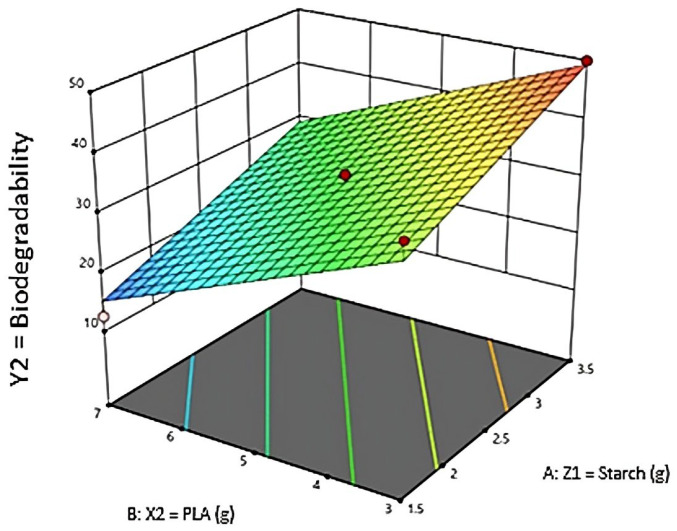
3D plot for biodegradability rate.

Biodegradation tests were conducted to determine the degradation rate of degradable plastics and determine the time it took for the plastic to decompose in the soil. Table 5 presents the validation data for the experimental and predicted response values determined by RSM. Table 6 shows the ANOVA for responses and presents the biodegradability test results of degradable plastics using RSM-CCD with design expert 13 software. Table 7 represents the statistical regression of biodegradability (%). From the research results that have been obtained, the effect of avocado seed starch and PLA on biodegradability (%) in degradable plastics will be discussed. Figure 3 presents the validation data set for experimental and predicted biodegradability of responses determined by RSM, meanwhile Figure 4 presents 3D plot for biodegradability rate.

### 3.3. Determination of Optimum Condition Result

Table 8 shows the parameters given for each independent and dependent variable. The optimal value for this study was derived from the parameters and boundaries for each variable, as presented in Table 9—this involved the optimization analysis of the water absorption and biodegradability variables.

### 3.4. Mechanical Properties Results

In this research, the material’s tensile strength was determined using a texture analysis tool. The tensile strength is maximized and can resist external forces before the degradable plastic is deformed or breaks. Breakage can occur by cracking due to over-stress or possibly by structural deformation. Table 10 shows the results from the tensile strength test with a weight variation of 1.8 g avocado seed starch and 3 g PLA.

### 3.5. Chemical Characterization Result

**Figure 5:**
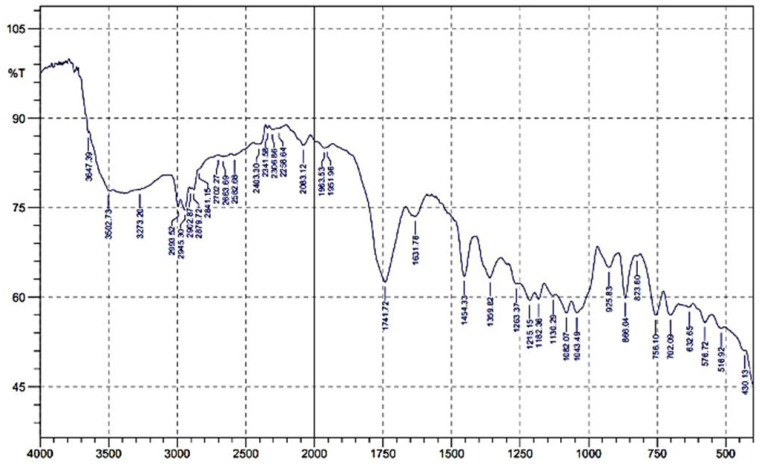
FTIR analysis of avocado-seed-starch-based degradable plastic and PLA.

Fourier-transform infrared spectroscopy (FTIR) was used in this study to determine the components of the materials used. The wavelength that the sample absorbs is characteristic of its molecular structure. FTIR analysis in this study was carried out at wave numbers 550–4000 cm^−1^. Table 11 and Figure 5 show the FTIR test on the degradable plastic sample based on avocado seed starch (1.8 g; PLA 3 g).

### 3.6. Thermal Properties Result

#### 3.6.1. Thermogravimetric Analysis (TGA) Result

**Figure 6:**
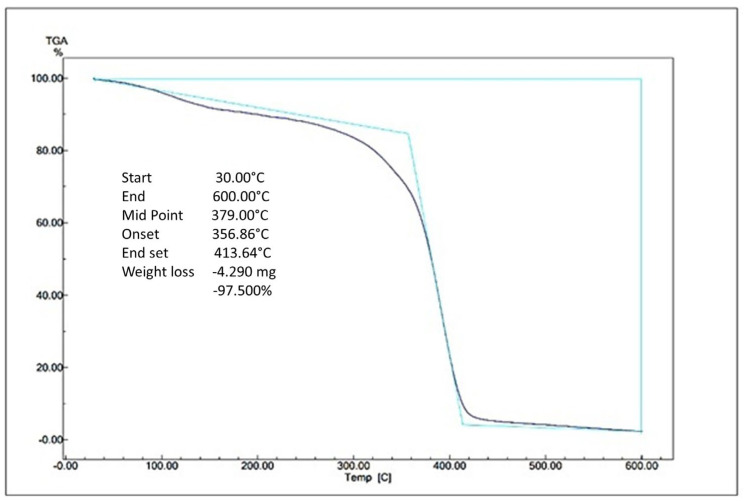
TGA of avocado-seed-starch-based degradable plastic and PLA.

The thermal stability of degradable plastics is obtained using the effects of polymer heating during physical changes (glass transition and melting) by Thermogravimetric Analysis (TGA). The TGA test was taken from the use of starch (1.8 g; PLA 3 g) in the degradable plastic. The curve generated in the TGA analysis is the change in mass vs. temperature, as shown in Figure 6.

#### 3.6.2. Differential Scanning Calorimetry (DSC) Result

**Figure 7:**
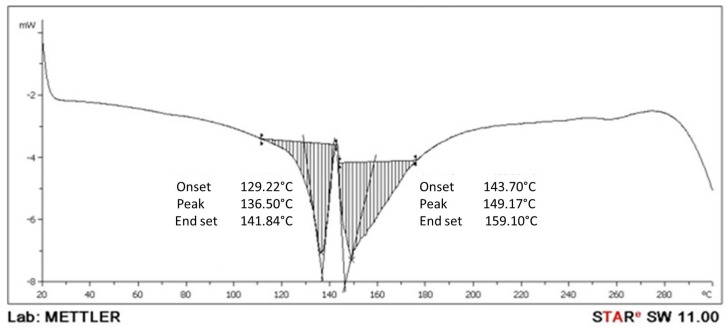
DSC of avocado-seed-starch-based degradable plastic and PLA.

The DSC technique allows for the determination of a polymer’s melting point, the temperature at which the crystalline phase begins to form, the temperature at which the mesomorphic transition occurs, the changes in enthalpy and entropy, the glass transition temperature, and other effects that feature either change in heat capacity or latent heat. The DSC test was conducted using starch (1.8 g; PLA 3 g) as the test component in the degradable plastic. The DSC curve can be seen in Table 12 and Figure 7.

#### 3.6.3. Melt Flow Rate (MFR) Result

**Figure 8:**
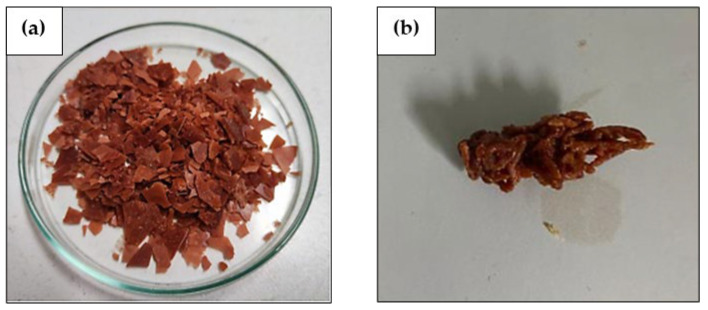
MFR analysis of avocado-seed-starch-based degradable plastic and PLA; (**a**) prior to testing and (**b**) after testing.

The melt flow rate (MFR) is a method of measuring the flow rate (melt) of polymer material in a specific unit of time (grams per ten minutes). This measurement is based on the specifications of the plastic material. Table 13 shows the melt flow rate test results of degradable plastics using avocado seed starch and PLA. The mass variation in both materials varied from the optimum condition (avocado seed starch 1.8 g and PLA 3 g). Figure 8 displays MFR analysis of avocado-seed-starch-based degradable plastic and PLA, prior and after testing.

### 3.7. Morphological Properties Result

**Figure 9:**
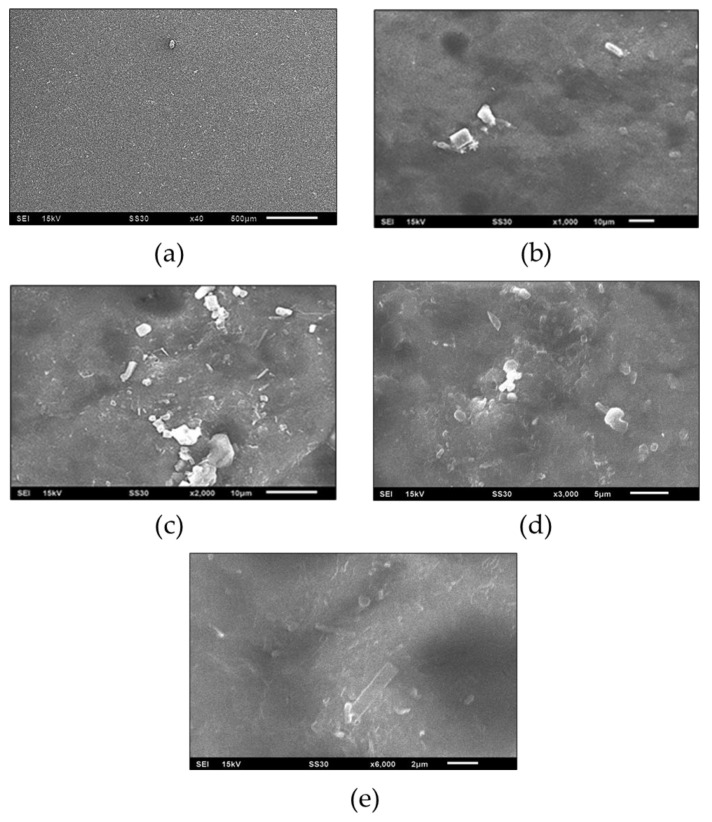
SEM analysis of avocado-seed-starch-based degradable plastic and PLA; (**a**) 40×, (**b**) 1000×, (**c**) 2000×, (**d**) 3000×, (**e**) 6000×.

A scanning electron microscope (SEM) is used to study a material’s surface, structure, and cross-section. It produces images of surfaces by scanning them with a focused beam of electrons, which interact with atoms on the surface. SEM can produce high-resolution images. The sample is observed in a controlled liquid environment. This study used the JEOL SEM with an acceleration voltage of 15 kV at 40×, 1000×, 2000×, 3000×, and 6000× magnification (1 area). SEM images of avocado seed starch 1.8 g using PLA 3 g for degradable plastic can be seen in Figure 9.

## 4. Discussion

### 4.1. Water Absorption Analysis

The correlation of the variables can be determined using the quadratic method. Table 2 shows that the water absorption analysis resulted in Equation (5) below.

Y_1_ = Equation Model (Process Order: Quadratic) is:

0.343404 + (0.592609 × X_1_) + (1.74201 × X_2_) − (1.15375 × X_1×2_) + (1.43500 × X_1_^2^) + (0.053125 × PLA^2^) (5)

In Table 3, an analysis of variance (ANOVA) was conducted to evaluate the effect of individual, quadratic, or interaction-dependent variables on the proposed model. The ANOVA results of the quadratic model in Table 2 were obtained based on the fit of all dependent variables to the quadratic model. If the probability value (*p* > F) for all coefficients is smaller than 0.05, then the coefficient is significant or has a natural effect on the experiment. In this case, X, Y, XY, and X2 is a significant model. However, if the probability value (prob > F) is more significant than 0.10, the variable or coefficient does not significantly affect the model. As carried out by Waday et al., (2023), the ANOVA results showed direct and interaction effects between the properties of orange peel oil and avocado seed starch, and the quadratic method was found to have a significant effect on the water vapor permeability value (*p* < 0.05) [23]. The 3D plots in Figure 2 above show that water absorption (%) is carried out to determine the ability of a degradable plastic to absorb water. So, the lowest water absorption is obtained at 1.5 g of starch and 3 g of PLA with an experimental value of 5.08% and an RSM-predicted value of 5.00%. At the same time, the highest optimal value for water absorption is 15.06% experiment value and 13.6% RSM prediction value with a composition of 3.5 g of starch and 3 g of PLA. The more starch added, the easier the resulting degradable plastic absorbs water. Tessanan et al., (2024) in their study analyzed the water resistance of degradable plastics from pineapple stem starch mixed with PLA with specific time intervals (1 to 30 days), resulting in a water absorption capacity that increases with the increase in immersion time and finally stops after a certain period [24]. Waday et al., (2023) used an Artificial Neural Network (ANN) and RSM modeling for the synthesis of avocado-seed-starch-based antimicrobial packaging with orange peel extract to determine the optimal value of water vapor permeability. The study showed that the models performed well, but the ANN-trained model had better modeling performance than the RSM [23].

Table 4, in the sum of the square test, a model is declared appropriate if the Adj R^2^ value and also Pred R^2^ have a value difference smaller than 0.2. When viewed from Table 4, the value of Adj R^2^ is 0.8326, and the value of Pred R^2^ is 0.3054, which indicates that this model is unsuitable because the difference between the values of Adj R^2^ and Pred R^2^ is more significant than 0.2. A model is also said to be good if the ratio of Adeq Precision is more than 4. The ratio of Adeq Precision on water absorption is 11.4307. ANOVA and 3D graphs were utilized to analyze the relationship between parameters and response. The coefficient of determination (R^2^) was used to rate the model’s fit to the data. Based on the statistical analysis of the models, all models are significant at a *p*-value lower than 0.05, the F-statistic is not significant, the Adj R^2^ value is close to the Pred R^2^ value, and the CV% value is below 10%. This indicates a strong correlation between the process factors and the relevant responses, and all models are repeatable [25].

### 4.2. Biodegradability Rate Analysis

Table 5 shows the results of Equation (6) for the biodegradability analysis. The calculation results from design expert V.13 provide a coefficient estimation model for each variable.

Y_2_ = Equation Model (Process Order: Quadratic) is:

39.64239 + (7.02925 × X_1_) − (4.99018 × X_2_) (6)

ANOVA is used to identify the significance of the model used in RSM—Table 6 shows the ANOVA results of the quadratic model obtained based on the fit of all dependent variables. If the probability value (prob > F) for all coefficients is smaller than 0.05, then the coefficient is significant or has a natural effect on the experiment. In this case, X, Y, XY, and X2 is a significant model. However, if the probability value (*p* > F) is more significant than 0.10, the variable or coefficient does not significantly affect the model. It can be seen from the 3D plots in Figure 4 that the biodegradability rate (%) of degradable plastics can be affected by starch and PLA. In this study, degradable plastic with a variation of starch 3.5 g and PLA 3 g is faster due to the many starches that accelerate the decomposition. After planting, the mass reduction rate is quite large, especially in the fifth sample, which is 49.9% in the experimental results and 49.3% in the RSM model prediction. The variation of starch 1.5 g and PLA 7 g took longer to degrade, with a range of 12.55% in the experimental results and 15.3% in the RSM model predictions. Zahri et al., (2021), using blended native Antarctic bacterial activity, selected environmental factors affecting the biodegradation of waste canola oil (WCO) and virgin canola oil (PCO), which were optimized using one-factor-at-a-time (OFAT) and RSM approaches. The obtained results using OFAT showed that the most effective microbial communities studied could degrade 94.42% and 86.83% within 7 days for WCO and PCO. While using RSM, 94.99% and 79.77% degradation could be achieved within 6 days. A significant interaction for RSM in biodegradation activity between temperature and WCO concentration in WCO media was shown [26].

Table 7, in the sum of square test, shows that a model is declared appropriate if the Adj R^2^ value and also Pred R^2^ have a value difference smaller than 0.2. The biodegradability result of Adj R^2^ 0.9351 and Pred R^2^ value 0.8882 have a difference value smaller than 0.2. A model is also said to be good if the ratio of Adeq Precision is more than 4. As seen in Table 7, the ratio of the Adeq Precision of biodegradability is 27.1221; this indicates an adequate model for this model is being used. Amaba et al., (2023) produced bioplastics from chitosan and mango seed starch (*Mangifera indica* L. Anacardiaceae) by a casting method using RSM. RSM was used to determine the effect of chitosan, glycerol, and starch on the biodegradability of bioplastics. The result was an optimal composition with a mass ratio of chitosan and starch of 1:0.17 (2% by volume of chitosan solution mixture) with 15.86% glycerol (per g of dry chitosan and starch) [27]. Degradable plastics standards (ASTM D6002-96 [28]: Guide for Assessing the Compostability of Environmentally Degradable Plastics and ASTM D20.96 [29] on Environmentally Degradable Plastics) state that, for products composed of a single polymer (homopolymer or random copolymer), 60% of the organic carbon has to be transformed into carbon dioxide by the end of the test period, i.e., a maximum of 180 days.

### 4.3. Determination of Optimum Conditions

The parameters for each independent and dependent variable are presented in Table 9. The desirability value represents the program’s ability to meet the criteria set for the final product. A desirability value close to 1 means the program can make a good product. Optimization aims to find the best conditions that bring together all the objective functions. A review of the optimization results in Table 10 reveals that the optimal response value is achieved when the independent variable levels are set at 1.894 g starch and 3.000 g PLA. The water absorption of the material was found to be 5.763%, and its biodegradability was calculated to be 37.988%.

Meanwhile, the desirability value obtained is 0.797, close to 1. The optimal conditions are found using 1.8 g starch and 3 g PLA. These ingredients analyze the strength of the plastic, its functional groups, its thermal properties, and its ability to resist water.

### 4.4. Tensile Strength, Elongation, and Young’s Modulus Analysis

This study conducted a tensile strength analysis of the optimal conditions of water absorption and biodegradability in the range of starch (1.8 g) and PLA (3 g). Mechanical properties were analyzed to determine the degradable plastics’ tensile strength, elongation, and modulus of elasticity values. Table 10 shows the tensile strength, elongation, and Young’s modulus value of 1.8 gr avocado seed starch using 3 gr PLA for degradable plastic. The mechanical strength value obtained in tensile strength was 10.127 MPa, elongation 85.75%, and Young’s modulus 190.02 MPa. Cao et al., (2023) optimized the process of making cow-dung- and hemp-fiber-based paper film using RSM with a Box-Behnken design, resulting in a tensile strength of about 8.26 MPa and a tear strength of about 19.91 N/mm. According to the study, further research is needed to obtain optimal mechanical results [30]. Tabassum et al., (2024) examined environmentally friendly composite films used as food packaging based on potato starch modified with kaolin and polyvinyl alcohol (PVA). This study resulted in a tensile strength of about 26.5 MPa, an elongation at a break of 96% at a 5.5 weight percent of kaolin, 2.5 g of starch, and 3.5 g of PVA [25]. Based on the Mat Web Material Property database for the category “Polypropylene, Extrusion Grade”, the tensile strength value in this study is almost comparable to the tensile strength obtained in HDPE (11.0–25.0 MPa) and PP (15.0–45.0 MPa). The stirring factor may affect the synthesis of degradable plastics to obtain optimum tensile strength. Cao et al., (2007) studied the fillers in pea starch plasticized with glycerol and water. In the range of 0 to 3 wt.% nanofiller, 2.85 to 4.73 MPa was obtained for tensile strength, 20.74 to 39.18 MPa for elastic modulus, and 41.99% (maximum) at 1 wt.% filler for elongation at break (if the filler increased above 1 wt.%, the elongation slightly decreased) [31]. In a study conducted by Anitha et al., (2024), the tensile strength of the bioplastic films they made was 4.25 and 2.35 MPa with elongation at a break of 39.5% and 25.4% for tapioca and sweet potato bioplastics [32].

According to Wojciechowska (2012), the elongation at break also increases with the increase in process temperature. In lower process temperatures, the molecules in the polymer matrix are compacted tightly with lower kinetic energy [33]. Based on the Mat Web Material Property database for the category “Polypropylene, Extrusion Grade”, the value of elongation at break is obtained in the range of 8–750% and modulus of elasticity at 0.680–3.60 GPa. The elongation and modulus of elasticity of avocado-seed-starch-based degradable plastics and the addition of PLA are comparable. Jade et al., (2022) made bioplastics by combining different types of starch (potato, tapioca, sago, and swamp taro) and protein. Tensile strength and elongation were obtained for potato (elongation 77.42%; tensile strength 2.52 ± 0.47 MPa), tapioca (elongation 165.25%; tensile strength 0.98 ± 0.13 MPa), sago (elongation 46.11%; 3.1 ± 0.56 MPa), and swamp taro (elongation 72.01%; 1.89 ± 0.48 MPa). Meanwhile, with 0.97 ± 0.13 MPa, tapioca has the lowest ultimate tensile, which means it will break and fracture faster than sago, with 3.09 ± 0.56 MPa. According to that study, starch–protein blend bioplastics made from sago and swamp taro are the most sustainable and promising for bioplastic production [34]. The material condition of amorphous glass and the presence of crystals significantly affect the mechanical response of bioplastics [35].

### 4.5. FTIR Analysis

In Figure 5, it can be observed that the wave numbers 3273.20 cm^−1^, 3502.73 cm^−1^, and 3647.39 cm^−1^ showed the presence of O-H stretching vibrations on the sodium hydroxide compound contained in starch. There are wavenumbers 2100–3600 cm^−1^, which are hydroxyl groups from starch, while, at wave numbers 2953.52 cm^−1^, 2945.30 cm^−1^, 2902.87 cm^−1^, and 2258.64 cm^−1^, there is a CH2 bond, the glucose content in avocado starch. Jiménez et al., (2022), in the study of making bioplastics based on Hass avocado seed starch, observed a peak wave number of 2919 cm^−1^, characteristic of CH bonds associated with methyl groups [36]. Wave numbers 1631.78 cm^−1^ and 1741.72 cm^−1^ are characterized as C=C and C-O stretching vibrations found in xylene–MA compounds and may also be related to PLA. The content of various functional groups can affect the inter-facial interface between starch and MA molecules in degradable plastics and may contribute to determining their mechanical and physical properties. As in the research of Navasingh et al., (2023), which involved making bioplastics based on rice starch and tapioca modified with calcium carbonate and plasticizers, there are peaks within 2883 cm^−1^ and 3000 cm^−1^, showing the presence of C-H stretching groups. The inclusion of starch has resulted in the presence of these groups. This would appear to resemble a sample that has been polymerized with glycerol. At a wave number of 1500, a N-H group of amines allows for an exothermic reaction between maleic anhydrous and sodium hydroxide in making degradable plastics [37].

Łopusiewicz et al., (2021), in their study on the preparation and bioactive characterization of composite films from CMC modified with fungal melanin and carvacrol, there are wave numbers 3200–3350 cm^−1^, which are hydroxyl groups from cellulose [38]. Gbadeyan et al., (2023), in their study, made bioplastics from snail shell and bagasse cellulose using PLA in FTIR analysis; the FTIR spectra showed a dominant interface of the C-O stretching, C=O stretching, and carboxylic acid-bending C-H stretching, showing the presence of bioplastic film functional groups [39]. According to Tan et al., (2022), for pure starch-based bioplastic films, there are wave numbers 3285.60 cm^−1^, 2926.23 cm^−1^, and 1648.86 cm^−1^, and for starch-based bioplastics mixed using chitosan reinforcement, there are wave numbers 3276.45 cm^−1^, 2925.32 cm^−1^, 1643.04 cm^−1^, and 1563.18 cm^−1^. He says the interaction and solubility between them is evidenced by the peak shift to lower wave numbers in FTIR [40].

### 4.6. Thermal Properties Analysis

#### 4.6.1. TGA Analysis

The TGA profiles of avocado-seed-starch-based and PLA degradable plastics are shown in Figure 6, respectively. There are weight losses in degradable plastic. The weight loss of the sample starts slowly at 30 °C. At this temperature, weight loss is caused by contaminants and other additives in the degradable plastic. The extreme weight loss starts at 356.86 °C to 413.64 °C, and crystallization occurs. In this state, most of the material decomposes and is completely depleted at 600 °C. In this study, the degradable plastic samples suffered a high level of thermal degradation. At this stage, the loss of hydrogen groups and the decomposition, depolymerization, and cracking of carbon chains in the starch structure occurred [41,42]. The total weight loss for avocado-seed-starch-based degradable plastic with PLA addition was 97.500% with 4.290 mg remaining. The thermogram change of TGA occurs due to the heat transfer of the degradable plastic and the reaction of the structure change and phase change of the degradable plastic. The significant weight loss in the range of 250–300 °C is due to the decomposition of gelatin and starch. From this, one could conclude that the prepared sample can be used in applications operating at high temperature levels [43].

López Terán et al., (2024) studied the effect of incorporating eucalyptus, tea tree (TT) rosemary essential oils, and chiriyuyo extract on the structure and properties of thermoplastic starch (TPS) obtained from potato starch, glycerin, and water. In the TGA analysis of the TT samples, the peaks shifted to lower temperatures with increasing TT concentrations (304.6, 301.6, 300.6, and 294.2 °C for TT concentrations of 0.5, 1.2, and 7 g/100 g TPS). According to them, increasing the concentration of tea trees in thermoplastic starch causes a significant change in the crystallinity of TPS [44]. It can be concluded from avocado-seed-starch- and PLA-based degradable plastics that the higher the decomposed residual weight, the better the thermal resistance.

#### 4.6.2. DSC Analysis

The DSC results are shown in Figure 7. There were two heats in this study’s DSC analysis, but this study observed it during the second heat. For this process, the heating is endothermic (the peak of the thermogram drops off). The peaks of the curves for avocado-seed-starch- and PLA-based plastics could be sharper. There is no glass transition, where the whole polymer is in a solid state at 149.17 °C, and the melting peak temperature is 159.10 °C. The melting process produces a peak on the DSC curve. Jozinovič et al., (2024) used DSC to analyze thermoplastic biopolymers made from potato starch and PLA. The curve peaked at 112.4 °C, indicating the cold crystallization temperature. PLA did not fully crystallize during heating, as shown by cold crystallization [45]. Calambás Pulgarin and Carolina Caicedo (2024) showed that biopolymers have two second-order transitions between 50 °C and 70 °C. Starch gelatinizes at a temperature of 64 °C when mixed with plasticizers. PLA has a glass transition temperature of 64 °C. However, the broadened band due to gelatinization overlaps with the PLA glass transition temperature, limiting comparative analysis [46]. In their study, Morales et al., (2023) determined that the melting point of pure PLA is 150 °C [47]. The findings illustrate that avocado-seed-starch-based degradable plastics, including PLA, exhibit a comparable melting point.

#### 4.6.3. MFR Analysis

MFR values are essential for determining material specifications, monitoring the quality of incoming raw materials, selecting an optimal processing method, and comparing the modified material to the original material to ensure that the requisite quality and specifications standards have been met. Molding capability is contingent upon the melt flow rate (MFR). The MFR is a critical parameter in the plastics industry [48]. Table 13 shows the MFR results of avocado-starch- and PLA-based degradable plastics with mass (starch 1.8 g and PLA 3 g). The values of degradable plastics cannot be quantified in terms of flow rate (melting) because the materials have already undergone a phase transition before measurement. This may be due to the temperature and load factors used in measuring the MFR, which are not at process conditions (190 °C/5 kg), but below. Introducing a starch–PLA blend alters the viscosity characteristics of the resulting degradable plastic. According to Dimonie et al., (2016), from research conducted, mixtures with a high starch content (70%) display a notable decrease in melt resistance to flow when the temperature is increased from 135 °C to 170 °C, particularly at loads of 2.16 kg and 3.8 kg. The slight enhancement in the melt’s ability to flow between 145 °C and 170 °C and under high loads (5 kg, 10 kg) may suggest cross-linking degradation. This process can elevate the molecular weight of the melt macromolecules [49]. Wang et al., (2021) studied thermoplastic starch/butyl glycol ester copolymer/polylactic acid (TPS/PBSA/PLA). As the PLA content increased, PLA and PBSA became incompatible, affecting the system’s performance. The MFR for TPS/PBSA/PLA composites decreased with PLA content, allowing for film blowing [50]. Table 14 presents the MFR results from previous studies.

Table 14 shows that blending PP and PE with TPS improves the MFR value of plastics. The MFR value of TPS blends with PP/PE is higher than the standard MFR value of PP/PE. This is due to the starch particles/granules that bind to the polymer matrix (PP/PE) with the addition of a compatibilizer. Upon examination via differential scanning calorimetry (DSC) analysis, the degradable plastic composite comprising avocado seed starch and PLA exhibited a melting point of 159.10 °C. This suggests that, in the context of prior research, the melt flow rate at 150 °C/5 kg, achieved through injection molding, is a probable process condition. Thus, degradable plastics such as household products, accessories, and so on can be molded as needed using injection molding machines. Thermoplastic starch serves as an ingredient that can increase the mixture’s viscosity. The viscosity value is also influential in determining the MFR value. The higher MFR value will affect the viscosity of the flow rate during the injection process [51].

### 4.7. SEM Analysis

The SEM analysis results demonstrate the morphology of the particles embedded within the matrix. It is thereby revealed whether the distribution of the particles within the matrix is homogeneous or heterogeneous. The SEM magnification of 40×, 1000×, 2000×, 3000×, and 6000× was used to analyze both avocado-seed-starch- and PLA-based degradable plastics, as demonstrated in Figure 9. It can be observed that the morphological results are more clearly visible at the 2000× magnification. The presence of white lumps and indentations on the surface of degradable plastics is evident in Figure 7. The results show that the plastic’s avocado seed starch PLA and other additives have not fully dissolved, so the shape is uneven. The main factor is stirring because it affects how well the starch PLA and additives dissolve. The surface features are comprised of granules (the residual portion of the starch particle), indicative of the incomplete gelatinization of the starch during its formation [55].

Micro-bubbles were observed on the surface of the degradable plastic made from the avocado starch and PLA blend. These micro-bubbles are caused by the hydrogen bonding chains of the starch, which begin to break down when the gelatinization temperature is reached, and water molecules begin to infiltrate the hydroxyl groups in the starch molecules [56]. For example, Zhang et al., (2021) conducted a study comparing PLA biofilms with those formed on conventional plastics. Their findings indicated that there were no significant differences between the two [57]. Cavities and micro-bubbles form when hydrogen bonds in long chains break during gelatinization. This lets water molecules into the hydroxyl groups of starch molecules [58]. The longer the stirring time, the better the solubility, while the faster the stirring time, the less soluble the components will be. The viscosity of the polymer affects the plastic’s morphology and density. Less viscous starch cannot retain vapor bubbles, and more dense starch cannot either [59]. Szatkowski et al., (2023) used PLA to make biocomposites with cracks from the bending stress test. The material broke when the PLA chain broke, and deep scratches appeared on the surface. The material could not take the force and could not be used [60].

## 5. Conclusions

The impact of avocado seed starch and PLA on degradable plastic degradation was investigated using RSM. Starch and PLA can influence degradable plastics’ water absorption and biodegradability rate. The results of this study show that 1.8 g and 3 g of PLA are the most optimal process parameters that also produce values that match the experimental results on water absorption and biodegradability rate. These parameters are used to analyze tensile strength, functional groups, and thermal and morphological properties. The mechanical characteristics of degradable plastics produced using 1.8 g avocado seed starch and 3 g PLA include a tensile strength of 10.127 MPa, elongation of 85.75%, and Young’s modulus of 190.02 MPa. In the FTIR analysis, the presence of organic groups was observed, and the degradable plastic was found to exhibit hydrophilic properties, indicating that it is susceptible to decomposition by soil. From the thermal properties of the TGA analysis results, degradable plastics have stable thermal resistance behavior. The DSC analysis revealed that the polymer exhibited no glass transition, maintaining a solid state at 149.17 °C, with a peak melting temperature of 159.10 °C. Further research is required to improve the accuracy of MFR analysis and ensure that the results align with the established standards for injection molding. It is crucial to ascertain the MFR value to facilitate the molding of degradable plastics by the desired specifications, as exemplified by the production of household products. In scanning electron microscopy (SEM), it was observed that the avocado seed starch, polylactic acid (PLA), and other additives did not fully dissolve in the plastic matrix, resulting in an uneven dispersion and an irregular surface. The primary factor influencing the dissolution of the ingredients is the stirring process.

The technology of the degradable plastic manufacturing process using the RSM method provides optimal results for operating conditions and the weight of the materials used so that it can be compared with commercial/conventional plastics, which will later be used as an application for household products that are safe for the environment and human health. This degradable plastic uses a natural base material obtained from avocado waste, namely the seeds, which can produce starch; this can increase the economic level for avocado farmers/gardeners and traders because the utilization of avocado seed waste has just been optimal. This opportunity is also expected to be one of the degradable plastic products to replace commercial/conventional plastics that impact environmental hygiene and human health.

## Figures and Tables

**Table 1:** RSM water absorption and biodegradability data using design expert software.

Run	Independent Variable		Dependent Variable	Dependent Variable
	X_1_: Starch (g)	X_2_: PLA (g)	Y_1_: Water Absorption (%)	Y_2_: Biodegradability (%)
1	2.5	7.82843	6.2	21.18
2	2.5	5	6.41	33.63
3	2.5	5	6.41	33.63
4	2.5	5	6.41	33.63
5	3.5	3	15.06	49.9
6	1.08579	5	7.41	19.27
7	3.91421	5	11.13	41.08
8	1.5	7	5.69	12.55
9	2.5	2.17157	7.45	42.77
10	2.5	5	6.41	33.63
11	1.5	3	5.08	38.25
12	3.5	7	6.44	26.29
13	2.5	5	6.41	33.63

**Table 2:** Validation data set for experimental and predicted water absorption values of responses determined by RSM.

Run	A: Starch (g)	B: PLA (g)	Water Absorption Experiment (%)	Water Absorption Predicted (%)	% Error
1	2.5	7.82843	6.2	5.1	1.1
2	2.5	5	6.41	6.4	0
3	2.5	5	6.41	6.4	0
4	2.5	5	6.41	6.4	0
5	3.5	3	15.06	13.6	1.5
6	1.08579	5	7.41	6.5	1.0
7	3.91421	5	11.13	12.1	1.0
8	1.5	7	5.69	7.1	1.5
9	2.5	2.17157	7.45	8.6	1.1
10	2.5	5	6.41	6.4	0.0
11	1.5	3	5.08	5.0	0.1
12	3.5	7	6.44	6.5	0.1
13	2.5	5	6.41	6.4	0.0

**Table 3:** ANOVA study for water absorption analysis responses.

Source of Water Absorption	Sum of Square	Df	Mean Square	F-Value	*p*-Value	
Model	79.54	5	15.91	12.93	0.0020	Significant
X_1_	31.96	1	31.96	25.99	0.0014	
X_2_	11.95	1	11.95	9.72	0.0169	
X_1_. X_2_	21.30	1	21.30	17.31	0.0169	
X_1_^2^	14.33	1	14.33	11.65	0.0112	
X_2_^2^	0.3141	1	0.3141	0.2554	0.6288	
Residual	8.61	7	1.23	-	-	
Lack of Fit	8.61	3	2.87	-	-	
Pure Error	0.0000	4	0.0000	-	-	
Cor Total	88.15	12	-	-	-	

**Table 4:** Statistical regression of water absorption (%).

Std. Dev.	1.11	R^2^	0.9023
Mean	7.42	Adjusted R^2^	0.8326
C.V.%	14.94	Predicted R^2^	0.3054
		Adeq Precision	11.4307

**Table 5:** Validation data set for experimental and predicted biodegradability values of responses determined by RSM.

Run	A: Starch (g)	B: PLA (g)	Biodegradability Experiment (%)	Biodegradability Predicted (%)	% Error
1	2.5	7.82843	21.18	18.2	−1.7938
2	2.5	5	33.63	32.3	−3.1928
3	2.5	5	33.63	32.3	−3.1928
4	2.5	5	33.63	32.3	−3.1928
5	3.5	3	49.9	49.3	−4.8875
6	1.08579	5	19.27	22.3	−2.2131
7	3.91421	5	41.08	42.2	−4.1795
8	1.5	7	12.55	15.3	−1.5130
9	2.5	2.17157	42.77	46.4	−4.5951
10	2.5	5	33.63	32.3	−3.1928
11	1.5	3	38.25	35.2	−3.4833
12	3.5	7	26.29	29.3	−2.9051
13	2.5	5	33.63	32.3	3192.8

**Table 6:** ANOVA study for biodegradability analysis responses.

Source of Biodegradability	Sum of Square	Df	Mean Square	F-Value	*p*-Value	
Model	1192.14	2	596.07	87.43	<0.0001	Significant
X_1_	395.28	1	395.28	57.98	<0.0001	
X_2_	796.86	1	796.86	116.89	<0.0001	
Residual	68.17	10	6.82			
Lack of Fit	68.17	6	11.36			
Pure Error	0.0000	4	0.0000			
Cor Total	1260.32	12				

**Table 7:** Statistical regression of biodegradability (%).

Std. Dev.	2.61	R^2^	0.9459
Mean	32.26	Adjusted R^2^	0.9351
C.V.%	8.09	Predicted R^2^	0.8882
		Adeq Precision	27.1221

**Table 8:** Parameters and boundaries for independent and dependent variables.

Name	Goal	Lower Limit	Upper Limit	Lower Weight	Upper Weight
Y_1_	In Range	1.5	3.5	1	1
Y_2_	In Range	3	7	1	1
Y_1_	Minimize	5.08	15.06	1	1
Y_2_	Maximize	12.55	49.9	1	1

**Table 9:** Optimization analysis.

X_1_: Starch (g)	X_2_: PLA (g)	Y_1_: Water Absorption (%)	Y_2_: Biodegradability (%)	Desirability
1.894	3.000	5.763	37.988	0.797

**Table 10:** Mechanical properties of avocado-seed-starch-based degradable plastic and PLA.

Type Sample	Tensile Strength (MPa)	Elongation (%)	Young’s Modulus (MPa)
Avocado-seed-starch-based degradable plastic and PLA Pure PLA	10.127 5.00–42.0	85.75 15.0–100	190.02 2960–3600

Pure PLA data are based on Matweb (material property data).

**Table 11:** Spektrum FTIR of avocado-seed-starch-based degradable plastic and PLA.

Bonding	Compound Type	Wave Numbers (cm^−1^)
O-H	Alcohol Monomer	3273.20, 3502.73, 3647.39
CH_2_	Alkana	2953.52, 2945.30, 2902.87, 2258.64
C=C	Alkena	1631.78
C-O	Carboxylic Acid	1741.72

**Table 12:** Summary of DSC analysis data on degradable plastics from avocado seed starch and PLA.

Area	Normalized (J/g)	Onset (°C)	End Set (°C)	Peak (°C)
I	−20.59	129.22	141.84	136.50
II	−29.93	143.70	159.10	149.17

**Table 13:** Melt mass flow rate test results.

Sample Name	MFR [g/10 min.]
Avocado-seed-starch-based degradable plastics + PLA	Not measurable. Sample melted before measurement.

**Table 14:** The MFR results from the previous research.

Sample Name	MFR Value	References
Sugarcane-bagasse-cellulose-based degradable plastics	[230 °C/5 kg] 1.02 ± 0.68 g/10 min	[48]
Corn-cob-cellulose-based degradable plastics	The sample was not measured, since it did not melt completely, rendering the measurement invalid.	[48]
Thermoplastic starch + polypropylene (TPS + PP)	[230 °C/2.16 kg] 10.9 ± 0.1 g/10 min	[51]
Thermoplastic starch + polyethylene (TPS + PE)	[190 °C/2.16 kg] 13.5 ± 0.1 g/10 min	[51]
Pure polypropylene (PP) using injection molding	[230 °C/5 kg] 5–20 g/10 min	[51]
Pure polyethylene (PE) using injection molding	[190°C/5 kg] 13–25 g/10 min	[51]
PP and PE standard value using compression molding	[230 °C/5 kg] 2 g/10 min	[51]
PP and PE standard value using blow molding	[190 °C/5 kg] 0.05–0.15 g/10 min	[51]
Poly(butylene succinate) (PBS) and Poly(butylene succinate-co-adipate) (PBSA)	[190 °C/0.325 kg] 3.6 ± 0.5 and 0.65 ± 0.01 g/10 min	[52]
PLA (material datasheet by Biomer for L9000)	3–6 g/10 min	[53]
Polypropylene (PP) standard value	[230 °C/5 kg] 1–5 g/10 min	[47,54]
Polyethylene (PE) standard value	[230 °C/5 kg] 1–3 g/10 min	[47,54]

## Data Availability

Data are contained within the article.
